# Urachal Adenocarcinoma: A Case Report of a Rare Tumor

**DOI:** 10.7759/cureus.69026

**Published:** 2024-09-09

**Authors:** Robin Okpara, Christopher Zias, Vishaal Kondoor, George Rodenko

**Affiliations:** 1 Radiology, Texas Tech University Health Sciences Center School of Medicine, Lubbock, USA; 2 Radiology, Medical Center Hospital, Odessa, USA

**Keywords:** adenocarcinoma, bladder, mucinous tumors, umbilicus, urachus

## Abstract

Urachal adenocarcinoma is a rare and aggressive bladder cancer involving the urachus, an embryological fibrous remnant of the allantois extending from the bladder to the umbilicus. Usually discovered in the advanced stages, this cancer can commonly present with a poor prognosis. We report a case of a 34-year-old male patient with an unremarkable medical history who presented to the emergency department with severe, sudden onset, sharp abdominal pain. Follow-up imaging revealed a tubular structure with mildly thickened and enhancing margins emanating from the anterior wall of the urinary bladder toward the umbilicus with peritoneal dystrophic small calcifications. These findings were susceptive to this rare tumor, and subsequent biopsy was indicative of a urachal mucinous adenocarcinoma.

## Introduction

The urachus is an embryological remnant of a channel between the bladder and the umbilicus; it typically involutes to become the median umbilical ligament, with the lumen obliterated in the third trimester [1]. However, in the case of partial involution, present in 32% of adults, numerous pathologies and potential malignancies can arise [2].

Urachal carcinoma is a rare and aggressive type of bladder cancer, accounting for <1% of all bladder cancers. This tumor is typically located in the anterior wall or bladder dome [1,3]. The most common type of urachal cancer is adenocarcinomas, accounting for >90%, considered to evolve from intestinal metaplasia of the epithelium [3]. The prognosis of this cancer is typically poor; symptoms do not commonly show early, leading patients to present at advanced stages, with local invasion or distant metastasis [4]. Because this cancer is so rare, there isn't a consensus on diagnostic criteria, staging, nomenclature, or therapeutic options. This case report describes a 34-year-old male patient who presents with primarily abdominal pain and is subsequently diagnosed with urachal adenocarcinoma.

This article was previously presented as a poster at the 2024 TTUHSC GSBS Annual Student Research Week on February 28, 2024.

## Case presentation

**Figure 1:**
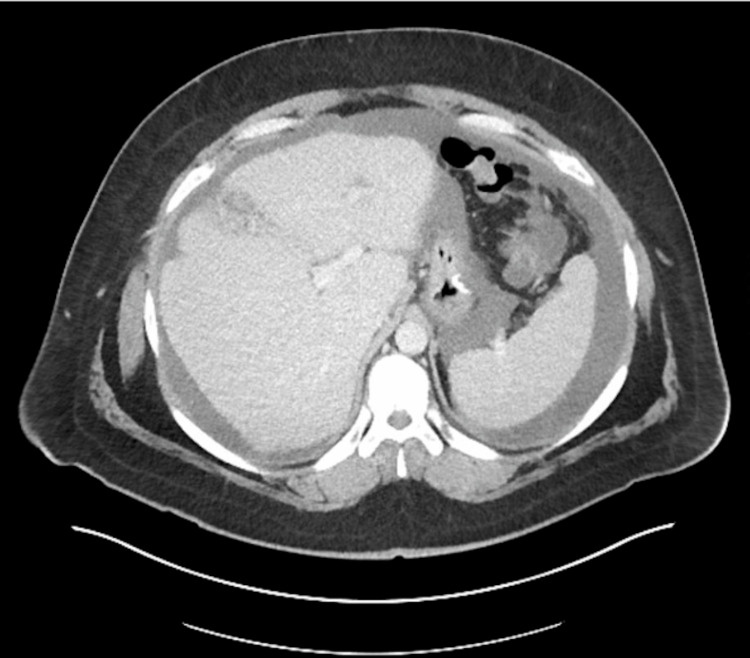
Large amount of peritoneal fluid in the abdominal cavity

**Figure 2:**
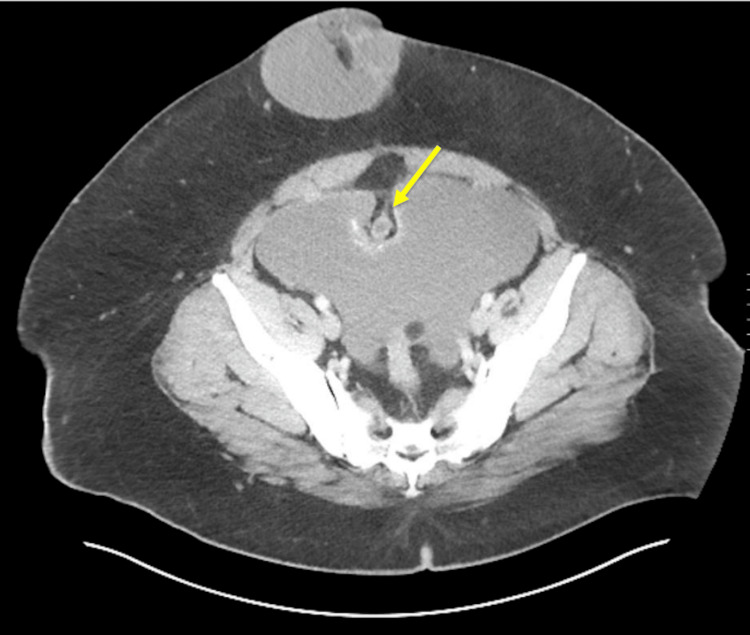
Peritoneal fluid with umbilical hernia. The urachal sinus tract indents the peritoneal membrane (yellow arrow)

The patient is a 34-year-old male who presented to the emergency department with severe, sudden onset sharp abdominal pain. An umbilical hernia and a pelvic mass were found at the site of pain. This hernia has been present for at least one year. His medical history was unremarkable, and he had no associated symptoms. An initial CT scan showed significant free fluid within the abdomen in addition to a large ventral hernia (Figures 1, 2). Physical exam findings showed tenderness to palpation at the umbilical site. A ventral hernia repair was recommended.

**Figure 3:**
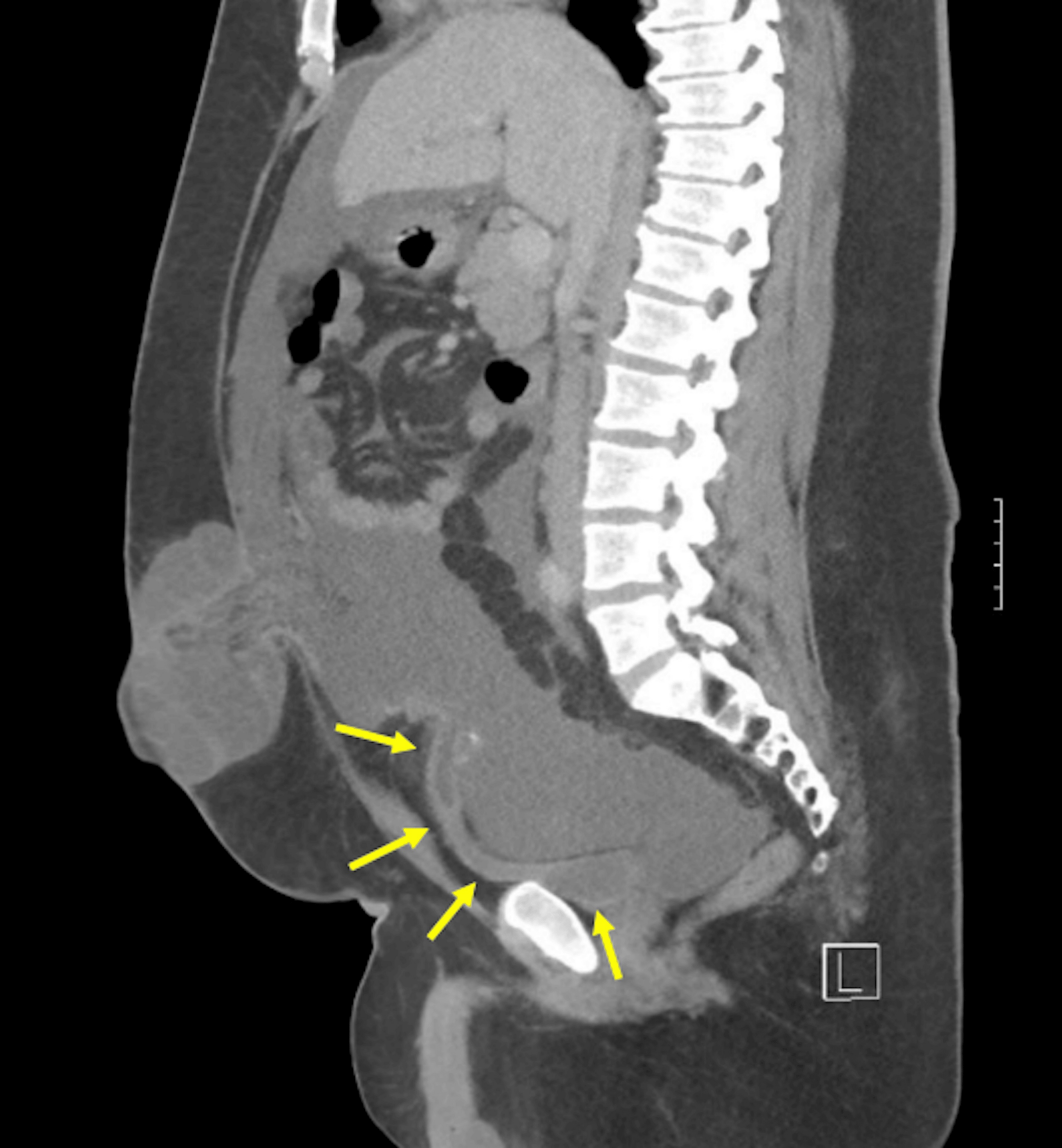
Urachal sinus tract from the urinary bladder toward the umbilicus (yellow arrows) with heterogenous calcifications

Repeat CT of the abdomen and pelvis revealed a tubular structure with mildly thickened and enhancing margins emanating from the anterior wall of the urinary bladder toward the umbilicus. In its superior aspect, there was a slightly greater area of fluid distention, with a surrounding wall and peritoneal dystrophic small calcifications (Figure 3). These findings were indicative of a urachal remnant, confirming the probability of the abdominal and pelvic malignancy being related to urachal carcinoma. In addition, very large amounts of ascites were present, as indicated in the previous scan.

An exploratory laparotomy was conducted, and findings included an incarcerated omentum and umbilical hernia with other findings of a small degree of abdominal ascites, a significant amount, i.e., greater than 3 L of gelatinous material, and a pelvic midline mass. A biopsy was conducted, and pathology showed well-differentiated urachal mucinous adenocarcinoma, confirming the patient’s CT findings. Oncology was consulted, a PET CT scan was ordered, and a one-month follow-up appointment was set. The patient was also referred to a tertiary center specializing in this rare cancer type.

## Discussion

The urachus, or median umbilical ligament, is an embryological remnant of the involution of the allantois and cloaca, extending from the umbilicus to the bladder dome. It is a tubular structure that typically obliterates with aging but persists in a small percentage of adults, about 30% [1,5]. Urachal tumors are rare and quite devastating, first described by Hue and Jacquin in 1863 [1,6]. They account for only 0.5% of all bladder malignancies and 20-40% of primary bladder adenocarcinoma [5]. While the urachus is lined by transitional epithelium, urachal carcinoma presents as adenocarcinoma in about 90% of cases [7].

Urachal adenocarcinoma is predominantly found in men in two-thirds of all cases, with a mean age of 60 (ranging from 40 to 70) [3]. The mean survival rate reported for locally advanced or metastatic disease is 12-24 months, and the five-year survival rate is roughly 43% [2]. The tumor is most commonly found in the portions adjacent to the dome of the bladder in 90% of cases [7]. In many cases, the tumor will extend superiorly to the umbilicus or inferiorly through the bladder wall. In rare cases, the tumor can be found in the middle of the urachus or closer to the end of the umbilicus [3].

Unfortunately, because early urachal tumors typically do not present with symptoms, patients commonly present with a higher stage, grade, and poor prognosis at the time of diagnosis [2,4]. Symptoms will show once the tumor has invaded the bladder wall, with hematuria being the most common, presenting in 90% of patients [2,8]. Other common symptoms include mucus-like discharge, dysuria, urinary pain, frequency, and urgency [3,9].

The diagnosis of urachal adenocarcinoma has been defined using criteria suggested by MD Anderson. The criteria consist of two main criteria and four supporting criteria. The two main criteria are the midline location of the tumor and a sharp demarcation between the tumor and normal surface epithelium. The four supporting criteria consist of enteric histology, the absence of primary adenocarcinoma of another origin, urothelial dysplasia, and cystitis cystica [1,2,10].

The standard imaging workup of urachal adenocarcinoma includes US, a CT scan, and/or MRI of the abdomen and pelvis. US is typically the first image of choice, and the tumor presents as a soft tissue mass that can have calcification and heterogeneity [2]. CT and MRI are more sensitive and are used for local staging and evaluation of distant metastasis. CT imaging can demonstrate the tumor's solid, cystic, or mixed features; solid and mixed are seen in 84% of cases [2,11]. Calcifications are a predominant feature found in adenocarcinomas of the abdomen, including urachal, present in about 50-70% of cases [3]. This can be seen as stippled, curvilinear, psammmomatous, or peripheral on CT [7]. MRI can show high-level intensity of T2-weighted images, possibly because of mucin content or necrosis [3]. T1-weighted images can show solid components of the tumor that may be isointense with soft tissue [8].

Currently, there is no standard adjuvant or metastatic chemotherapy protocol for treating urachal adenocarcinoma [1]. The role of radiation and chemotherapy and their benefits to patients have remained unclear. Surgical resection, specifically the open surgical approach, is the mainstay therapy. There is a lack of long-term data on laparoscopic or robotic surgeries [1,9].

## Conclusions

While urachal adenocarcinoma is a rare cancer, there are specific findings on imaging to confidently suggest the diagnosis before a biopsy is conducted. Visualizing a soft tissue midline pelvic mass extending from the bladder dome to the umbilicus highly suggests urachal adenocarcinoma. Additionally, calcifications present in the mass are an additional factor that will strengthen diagnosis. It is essential to diagnose this cancer as soon as possible, as it commonly will present in advanced stages, where the patient has a lot of symptoms. Treatment will need to be guided and weighed with what is best for the patient; this could be chemotherapy or radiation, surgical resection, or partial or complete cystectomy.
